# Relationship between ruminative dispositions and perceived sports performance in young elite athletes in Hong Kong: the role of problem-oriented coping strategies

**DOI:** 10.3389/fspor.2025.1513277

**Published:** 2025-02-28

**Authors:** Dong-Tai Chen, Jui-Ti Nien, Xiaoling Geng, Jeffrey Yu, Chatkamon Singhnoy, Yu-Kai Chang

**Affiliations:** ^1^ Department of Physical Education and Sport Sciences, National Taiwan Normal University, Taipei, Taiwan; ^2^ Department of Sports Performing Arts, University of Taipei, Taipei, Taiwan; ^3^ Faculty of Sport Science, Burapha University, Saen Suk, Thailand; ^4^ Social Emotional Education and Development Center, National Taiwan Normal University, Taipei, Taiwan

**Keywords:** multi-dimensional rumination, repetitive thinking, stress management, sports performance, athletic performance

## Abstract

There is a nuanced relationship between rumination and sports performance, which may depend on individuals being predisposed to specific facets of rumination. Additionally, ruminative dispositions are intertwined with coping strategies, with both playing crucial roles in sports performance. This study aimed to investigate the relationships among ruminative dispositions, coping strategies, and sports performance in athletes, considering the perspective of multi-dimensional rumination. This study also examined whether coping strategies are associated with the relationship between ruminative dispositions and perceived sports performance. Parallel mediation analysis was conducted on 111 young elite athletes from the Hong Kong national team to examine the relationships between ruminative dispositions, coping strategies, and sports performance. The results revealed that emotion-focused ruminative disposition (ERD) and meaning-searching ruminative disposition (MRD) were negatively associated with perceived sports performance, with problem-oriented coping (POC) playing a partial role. In contrast, instrumental ruminative disposition (IRD) was positively associated with perceived sports performance, fully via POC. These findings suggest that athletes with higher levels of ERD and MRD tend to use POC less frequently, which was associated with poor perceived sports performance. Conversely, athletes with higher levels of IRD tend to employ POC more frequently, which was positively associated with perceived sports performance. The proposed model provides the theoretical framework for multi-dimensional rumination in sports psychology and outlines the potential impact of coping strategies on athletic performance. Importantly, this research underscores that the outcome of rumination is contingent upon its focus.

## 1 Introduction

Rumination, the cognitive process of repetitive thinking about a specific experience (1), often involves personal dissatisfaction or immediate distress (2, 3). According to the Goal Progress Theory (4), rumination is triggered when an athlete's performance falls below the expected goal (e.g., making a mistake in competition) and continues until the performance gap is diminished or the athlete adjusts the initial goal (5, 6). Rumination may lead to both negative and positive outcomes in performance. For instance, rumination has been shown to negatively predict problem-solving performance through increased perceived stress and negative mood, but also positively predict problem-solving performance through increased attention and effort (7). Most research related to sports performance has focused on the negative aspects of rumination (e.g., dwelling on negative emotions or poor decisions), particularly its association with an impaired ability to address stressors in competition (8), suboptimal accuracy in basketball passing (9), and increased unforced errors in tennis (10). These findings highlight the need for a more thorough investigation into the relationship between different categories of rumination and sports performance.

The consequences of rumination are associated with the individual's ruminative dispositions (11), and within the sports context, a common classification of rumination distinguishes between “brooding rumination” (also referred to as “rumination”) and “reflective rumination” (also referred to as “reflection”) (12–18). Brooding ruminators more often concentrate on unconcreted elements, such as emotions, rather than the problems themselves, while reflective ruminators tend to focus on problems themselves, such as identifying the causes and exploring potential solutions (19, 20). A three-wave longitudinal study that aimed to discriminate adaptive and maladaptive forms of ruminative dispositions eventually categorized brooding rumination as a maladaptive form and reflective rumination as an adaptive form (21). However, it should be noted that even reflective rumination, which was considered a relatively more adaptive form than brooding rumination, may still be associated with maladaptive outcomes. A recent meta-analysis indicated that worry not only positively correlated to brooding rumination, but also to reflective rumination (22). In the sports context, Roy et al. (18) found that higher-level athletes demonstrate a lower degree of both brooding and reflective rumination. Therefore, it may be an oversimplification to regard reflective rumination solely as adaptive, and a more nuanced examination of the processes underlying reflective rumination and its consequences within the sports context is warranted.

Specifically, reflective rumination can be further categorized into “abstract reflection,” which involves exploring the root causes of problems (i.e., focusing on attribution), and “concrete reflection,” which focuses on devising strategies to solve or prevent similar problems (i.e., focusing on problem-solving) (20, 23). An empirical study in psychiatry found that a ruminative disposition characterized by a focus on problem-solving significantly predicted a reduction in depression symptoms over the following 5 weeks, whereas a ruminative disposition characterized by a focus on attribution did not (24). In the sports context, research on athlete ruminative dispositions has primarily focused on two-factor models, distinguishing between brooding and reflective rumination (16–18) or focused solely on brooding rumination (12–15). However, given that the focus of rumination may influence its outcomes, adopting a comprehensive framework that considers distinct aspects of ruminative dispositions, such as emotion, attribution, and problem-solving, within the sports context is crucial.

Previous studies have proposed a multi-dimensional framework for ruminative dispositions, dividing it into three dimensions (25, 26). Emotion-focused ruminative disposition (ERD) is characterized by continuous immersion in the emotion of negative experiences (25). Meaning-searching ruminative disposition (MRD) focuses on seeking the ultimate reason for a negative event (25). Instrumental ruminative disposition (IRD) involves the repetitive consideration of solutions and prevention strategies (25). Based on these characteristics, ERD closely resembles brooding rumination, MRD aligns closely with abstract reflection, and IRD is closely related to reflection. In the examination of patients with coronary heart disease, Fritz (25) found that ERD was positively correlated with the severity of mood disturbance at admission and 4 months post-hospitalization, and was negatively correlated with mental functioning 4 months post-hospitalization. MRD was positively correlated with the severity of mood disturbance 4 months post-hospitalization, whereas IRD was negatively correlated with the severity of mood disturbance at admission (25). These results suggest that focusing on emotion and attribution in ruminative dispositions (i.e., ERD and MRD) may lead to maladaptive outcomes, whereas focusing on problem-solving (i.e., IRD) may be adaptive (25, 26). To our knowledge, in a sports context, only Wu et al. (27) reported that mindfulness training simultaneously decreases ERD and increases archery performance, suggesting a potential association between multi-dimensional rumination and specific performance. More studies directly testing this relationship are warranted.

The relationship between ruminative dispositions and sports performance may also be explained through the utilization of coping strategies. Coping refers to the cognitive and behavioral responses individuals employ to manage current stressful events in response to environmental or personal demands (28, 29). Carver et al. (30) classified coping strategies into approach-orientation and avoidance-orientation based on individual responses to stressors, where approach-orientation further is divided into problem-oriented coping (POC) and emotion-oriented coping (EOC). POC involves actively seeking solutions to address stressors, whereas EOC aims to alleviate emotional experiences associated with the stressor (31, 32). Avoidance-oriented coping (AOC) involves denying stressors, engaging in alternative activities to escape stressful events, or ceasing efforts to reduce perceived stress rather than addressing them directly (31, 32).

Athletes' peak performance in competition hinges on their ability to adopt effective coping strategies (33). A meta-analysis indicated that athletes who employed coping strategies involving task-oriented and problem-focused engagement and an approach to taking control of stressors experienced a positive effect on sports performance; conversely, athletes who utilized coping strategies involving ceasing efforts toward goal attainment may experience negative outcomes (34). These findings support the hypothesis that POC may be positively associated with sports performance, while AOC may be negatively associated. Although there is no meta-analytical evidence directly supporting the association between EOC and performance, the regulation of stress-related emotions (e.g., anxiety and anger) when confronting stress is used more often and is crucial for sports performance (35, 36). Several studies have indicated that various emotion regulation approaches can improve sports performance (37–40). Additionally, successfully regulating emotions after a sports performance that was below expectations can lead to subsequent improvements in sports performance (41). These findings highlight the potential of EOC in optimizing sports performance.

Ruminative dispositions are also associated with coping strategies. Burwell and Shirk (42) found that adolescents who focused on problem-solving were likely to employ POC and EOC, whereas those who ruminated on emotional content tended to use AOC. In university students, another study found that those who focused on their own emotions during rumination were more likely to employ AOC in response to stress (43). Tan et al. (44) found similarities in a study on caregivers of breast cancer patients, further indicating that using AOC to deal with stressful events not only failed to address emotional and stress-related consequences but also increased feelings of stress and anxiety. Above all, individuals with higher ERD are less likely to use POC and EOC to handle stressors and instead prefer to employ AOC. In contrast, individuals with higher IRD are more likely to use POC and EOC and less likely to employ AOC. Although few studies have explored the relationship between MRD and coping strategies, the multi-dimensional framework for ruminative dispositions suggested that individuals with higher MRD or ERD may lead to negative consequences (25, 26, 45), indicating a potentially similar relationship with coping strategies as observed in ERD. However, a gap exists in sports research, with Josefsson et al. (8) being the only study to identify a negative association between emotion-focused rumination and coping efficacy. Further studies exploring the multi-dimensional relationship between ruminative dispositions and coping strategies are warranted.

The current study aimed to explore the relationship between multi-dimensional ruminative dispositions and perceived sports performance, and examine whether the type of coping strategy serves as a connection that makes an indirect relationship between multi-dimensional ruminative dispositions and perceived sports performance. It was hypothesized that maladaptive ruminative dispositions (i.e., ERD and MRD) would be negatively associated with perceived sports performance, involving more avoidance-orientation (i.e., AOC) and less approach-orientation (i.e., POC and EOC). Additionally, an adaptive ruminative disposition (i.e., IRD) was expected to be associated with perceived sports performance, involving more approach-orientation (i.e., POC and EOC) and less avoidance-orientation (i.e., AOC).

## 2 Methods

### 2.1 Participants

This study recruited active elite athletes from various sports representing the Hong Kong national team. The recruitment process began by contacting the head coaches of various sports within the Hong Kong national team via email to obtain approval. Following the initial contact, the researcher visited all teams in person twice. During the first visit, the researcher explained the study to the entire team, detailing the purpose, procedures, and potential risks. Informed consent forms following the Declaration of Helsinki were provided to all the athletes, who were informed of their right to withdraw from the study at any time and without negative consequences. They were also assured that their responses and personal information would be kept confidential and not disclosed to coaches, teammates, or others. Subsequently, the athletes returned the signed informed consent forms during the second visit, which was particularly important for those under 18 years old who required parental or guardian approval. The second visit focused on data collection through paper questionnaires. Only athletes who provided written informed consent were formally enrolled in the study and included in the data collection phase. Athletes completed the surveys independently and returned them to the researcher immediately upon completion. Notably, the surveys were administered anonymously, as participants did not sign their names before returning the questionnaires.

The data screening process involved the exclusion of questionnaires with duplications, missing responses, or unclear answer options. Subsequently, samples suspected of exhibiting response style were removed (46). Specifically, this included those who exhibited characteristics of extreme responding, middle-point responding, acquiescence or criticalness, and random responding (46, 47). Additionally, participants who failed to adhere to the instructed-response items were excluded from the analysis (47, 48).

The final dataset for analysis comprised questionnaire responses from 111 young elite athletes (*M_age_* = 19.57 years, *SD_age_* = 5.76, including 55 male athletes and 56 female athletes), including badminton (*n* = 16), swimming (*n* = 13), martial arts (*n* = 50), squash (*n* = 9), bowling (*n* = 4), fencing (*n* = 5), track and field (*n* = 2), gymnastics (*n* = 3), volleyball (*n* = 1), rowing (*n* = 5), and cycling (*n* = 3). They have all achieved at least one of the following within the past four years: a top-three finish at national games or a top-eight finish at international games in their specialty, either in age-based or open-age categories.

### 2.2 Measures

#### 2.2.1 Ruminative dispositions

The Chinese version of the Multi-Dimensional Rumination Scale [CMDRS; (45)] was used and was adapted from the Multi-dimensional Rumination Scale (25). The CMDRS comprises three dimensions: ERD with 13 items (e.g., “How often do you only think about your negative feelings in sports?”), MRD with 7 items (e.g., “How often do you think about why things don't turn out the way I expect them to in sports?”), and IRD with 5 items (e.g., “How often do you come up with strategies to solve problems that occur in sports?”), totaling 25 items. Scoring was conducted using a Likert five-point frequency scale from 1 (almost never) to 5 (almost always), with higher scores indicating a stronger inclination towards a specific ruminative disposition. Additionally, to align the scale descriptions more closely with the sports context, the text “in sports contexts such as during training and competition” was incorporated into item descriptions (27).

The CMDRS has demonstrated good construct validity and internal consistency in college students (45) and good internal consistency in young athletes (27). Similarly, in this study, the internal consistency for each construct was good (ERD: McDonald's *ω* = .958; MRD: McDonald's *ω* = .855; IRD: McDonald's *ω* = .892), as was that for the overall scale (McDonald's *ω* = .956).

#### 2.2.2 Coping strategies

The Chinese version of the Athletic Coping Strategies to Problems Experienced Scale [A-COPE; (49)], which is a modification of the Coping Orientation to Problems Experienced Scale [COPE scale; (30)] was used. Furthermore, A-COPE was adapted to include descriptions tailored to sports-related situations. A-COPE comprises three dimensions: POC with 15 items (e.g., “I consider the best ways to address the challenges of participating in sports.”), EOC with 9 items (e.g., “I try to get emotional support from teammates, coaches, or close family members.”), and AOC with 5 items (e.g., “I admitted I couldn't handle it before even trying in sports.”), totaling 29 items. Scoring was conducted using a Likert seven-point frequency scale from 1 (almost never) to 7 (almost always), with higher scores indicating a greater tendency for individuals to use specific coping strategies aligned with a particular orientation. Similarly, the phrase “such as during training and competition” was added after “sports” in all items. The A-COPE in young athletes has demonstrated well-established construct validity, criterion validity (49), and internal consistency (49, 50). Similarly, in the current study, the internal consistency for each construct was good (POC: McDonald's *ω* = .877; EOC: McDonald's *ω* = .855; AOP: McDonald's *ω* = .797), as was that for the overall scale (McDonald's *ω* = .911).

#### 2.2.3 Perceived sports performance

Perceived sports performance over the past month was measured using a single-item Likert scale (51–53). Participants were asked to rate their performance on a scale of 1 (bad) to 10 (perfect), with higher scores indicating a greater perception of performance being favorable (54). This approach has been widely utilized in various studies (51–55). Furthermore, it is considered an appropriately standardized measurement across diverse specialties for elite athletes (51). To ensure meaningful ratings from off-season participants, we employed an integrated assessment approach. Participants were asked to consider their performance in both training and competition contexts and respond to the question (i.e., “How do you think about your performance in sports such as training and competition?”).

### 2.3 Statistical analysis

Data analyses were conducted using JASP version 0.16.3 and SPSS version 25.0. JASP was employed to analyze the internal consistency index (i.e., McDonald's *ω*) (56) of each dimension and the scales overall. SPSS was utilized to compute the descriptive statistics, including means (*M*) and standard deviations (*SD*) for each variable.

Pearson correlation analyses were performed using SPSS to assess the strength of the associations between coping strategies [mediators (MEs)] and both ruminative dispositions [independent variables (IVs)] and perceived sports performance [dependent variable (DV)]. Furthermore, the strength of the associations between the three ruminative dispositions (ERD, MRD, and IRD) was explored (Supplementary Table 1). Effect size strengths (i.e., correlation coefficients) of 0.1, 0.3, and 0.5 were regarded as small, medium, and large, respectively (57). To control the family-wise error rate associated with multiple correlations, the significance level for the Pearson correlation analyses was adjusted downward (58). Following Cupples et al. (59), the significance level was reduced from *α* = .05 to *α* = .013 in the correlations between each ME and both IVs and the DV. It was also reduced from *α* = .05 to *α* = .017 in correlations among the three ruminative dispositions (see Supplementary Figure 1 for the correction formula). Independent samples *t*-tests in SPSS were then conducted to examine the differences in ruminative dispositions, coping strategies, and perceived sports performance between sexes (male vs. female) and developmental stages (Supplementary Tables 2, 3). We categorized the participants into two developmental stages [adolescence (aged ≤19 years old) vs. adult (aged >19 years old) (60)]. The significance level for the *t*-tests was set at *α* = .05.

For the parallel mediation analysis, SPSS PROCESS macros Model 4 (61) were employed. Prior to analysis, sex was coded as a dummy variable, with male athletes represented as 0 and female athletes as 1. Continuous variables were standardized into *z*-scores to standardize all regression coefficients (62). Coping strategies (i.e., POC, EOC, and AOC) were considered mediators in the relationship between ruminative dispositions (i.e., ERD, MRD, and IRD) and perceived sports performance. Bootstrapping with 5,000 resamples was used. The determination of the mediation model's validity was based on the 95% confidence intervals (CIs) of the indirect associations. Significance was considered when the 95% CI did not include zero (63). For the power calculation, which was difficult to estimate from a previous study, a “*post hoc*” power analysis (64) using the Monte Carlo Power Analysis for Mediation Models was conducted (65).

## 3 Results

### 3.1 Correlation analysis

For our medium-sized sample (*n* = 111), the data for each continuous variable were standardized into *z*-scores and none fell outside the ±3.29 range, further supporting the absence of outliers (66, 67). Moreover, we examined the skewness and kurtosis *z*-scores to assess normality (i.e., |*z*| < 3.29) (68, 69). The variables in the correlation analysis (i.e., ruminative dispositions, coping strategies, and perceived sports performance) met this criterion. Additionally, the dichotomous variable (i.e., sex) exhibited approximate uniform distribution (male athletes = 55 and female athletes = 56). The descriptive statistics for all the variables are presented in Table 1.

**Table 1:** Descriptive statistics among the variables (*N* *=* 111).

Variable	*M*	SD	95% CI	*Z*_skewness_	*Z*_kurtosis_
Control variables					
1. Age	19.568	5.757	[20.640, 18.500]	2.834	−0.182
2. Sex	0.505	0.502	[0.599, 0.411]	–	–
3. WTR	22.126	8.374	[23.684, 20.568]	−3.459	−0.099
Ruminative dispositions					
4. ERD	2.355	0.833	[2.510, 2.200]	1.445	−0.796
5. MRD	2.270	0.804	[2.420, 2.120]	1.441	−1.497
6. IRD	3.620	0.603	[3.732, 3.508]	0.066	0.167
Coping strategies					
7. POC	4.622	0.826	[4.776, 4.468]	0.755	−0.011
8. EOC	4.741	0.991	[4.925, 4.557]	1.301	−0.927
9. AOC	2.344	0.959	[2.522, 2.166]	3.022	−0.086
Perceived sports performance	6.153	1.701	[6.469, 5.837]	−2.738	0.055

WTR, weekly training hours; ERD, emotion-focused ruminative disposition; MRD, meaning-searching ruminative disposition; IRD, instrumental ruminative disposition; POC, problem-oriented coping; EOC, emotion-oriented coping; AOC, avoidance-oriented coping. Sex: male = 0, female = 1.

The associations between coping strategies and both ruminative dispositions and perceived sports performance are presented in Table 2. POC showed a significant negative correlation with ERD [*p* = .008, 95% CI (−0.417, −0.067)], a non-significant correlation with MRD [*p* = .035, 95% CI (−0.373, −0.014)], and a significant positive correlation with both IRD [*p* < .001, 95% CI (0.466, 0.707)] and perceived sports performance [*p* < .001, 95% CI (0.303, 0.598)]. EOC showed a significant negative correlation with ERD [*p* = .003, 95% CI (−0.445, −0.101)], a non-significant correlation with MRD [*p* = .048, 95% CI (−0.362, −0.002)], and a significant positive correlation with both IRD [*p* < .001, 95% CI (0.172, 0.501)] and perceived sports performance [*p* < .001, 95% CI (0.161, 0.493)]. AOC showed a significant positive correlation with ERD [*p* < .001, 95% CI (0.425, 0.681)] and MRD [*p* < .001, 95% CI (0.419, 0.677)], while showing a non-significant correlation with IRD [*p* = .153, 95% CI (−0.315, 0.051)] and a non-significant correlation with perceived sports performance [*p* = .170, 95% CI (−0.310, 0.057)].

**Table 2:** Correlation matrix of ruminative dispositions, coping strategies, and perceived sports performance.

Variable	POC	EOC	AOC
Ruminative dispositions			
ERD	−.250**	−.282**	.567***
MRD	−.200*	−.188*	.562***
IRD	.600***	.348***	−.137
Perceived sports performance	.463***	.337***	−.131

ERD, emotion-focused ruminative disposition; MRD, meaning-searching ruminative disposition; IRD, instrumental ruminative disposition; POC, problem-oriented coping; EOC, emotion-oriented coping; AOC, avoidance-oriented coping.

* *p* < .05.

** *p* < .01.

*** *p* < .001.

### 3.2 Parallel mediation analysis

#### 3.2.1 The ERD and perceived sports performance model

**Figure 1:**
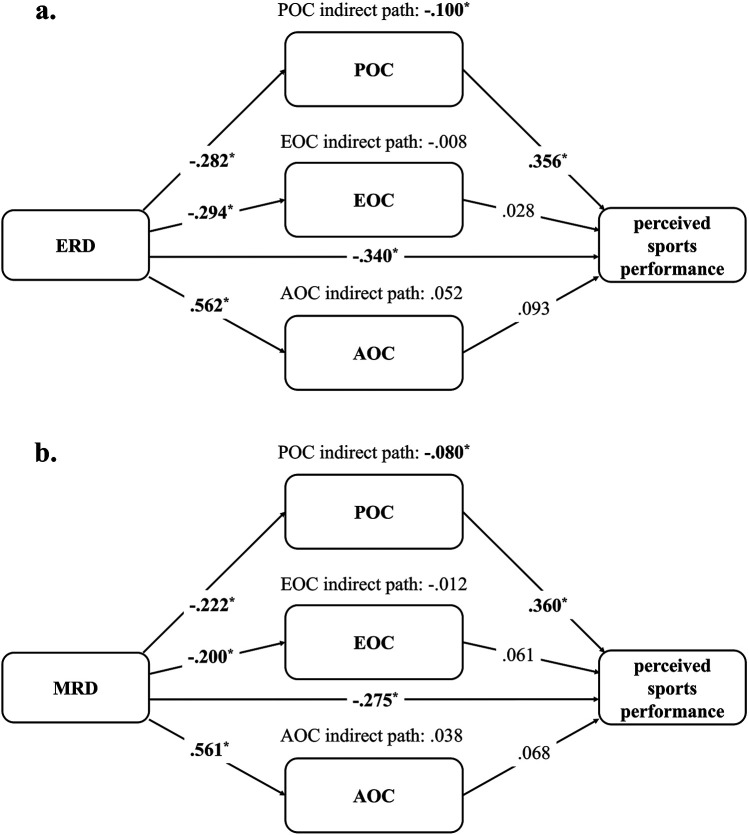
The models of maladaptive ruminative dispositions and perceived sports performance. **(a)** Emotion-focused ruminative disposition and perceived sports performance model. **(b)** Meaning-searching ruminative disposition and perceived sports performance model. Age, sex, and weekly training hours were control variables. ERD, emotion-focused ruminative disposition; MRD, meaning-searching ruminative disposition; POC, problem-oriented coping; EOC, emotion-oriented coping; AOC, avoidance-oriented coping. All the indexes are standardized regression coefficients (*β*); ^*^ CI does not include zero.

After controlling for age, sex, and weekly training hours, the indirect association through POC was significantly negative [95% CI (−0.027, −0.208)], while EOC [95% CI (0.078, −0.078)] and AOC [95% CI (0.177, −0.042)] were non-significant. Concurrently, ERD had a significantly negative direct association with perceived sports performance [95% CI (−0.137, −0.542)]. Therefore, the model indicated that only POC partially mediated the relationship between ERD and perceived sports performance (Figure 1a).

Specifically, regarding each of the associations within the model, ERD was significantly negatively associated with POC [95% CI (−0.094, −0.470)] and EOC [95% CI (−0.105, −0.482)], while it was significantly positively associated with AOC [95% CI (0.724, 0.400)]. Among the coping strategies, only POC was significantly positively associated with perceived sports performance [95% CI (0.583, 0.128)], while EOC [95% CI (0.256, −0.201)] and AOC [95% CI (0.289, −0.103)] were not (Figure 1a).

#### 3.2.2 The MRD and perceived sports performance model

After controlling for age, sex, and weekly training hours, the indirect association through POC was significantly negative [95% CI (−0.012, −0.164)], while EOC [95% CI (0.037, −0.069)] and AOC [95% CI (0.162, −0.061)] were non-significant. Simultaneously, MRD had a significantly negative direct association with perceived sports performance [95% CI (−0.073, −0.477)]. Therefore, the model indicated that only POC partially mediated the relationship between MRD and perceived sports performance (Figure 1b).

Specifically, regarding each of the associations within the model, MRD was significantly negatively associated with POC [95% CI (−0.032, −0.412)] and EOC [95% CI (−0.009, −0.392)], while it was significantly positively associated with AOC [95% CI (0.722, 0.400)]. Among the coping strategies, only POC was significantly positively associated with perceived sports performance [95% CI (0.592, 0.129)], while EOC [95% CI (0.293, −0.171)] and AOC [95% CI (0.269, −0.133)] were not (Figure 1b).

#### 3.2.3 The IRD and perceived sports performance model

**Figure 2:**
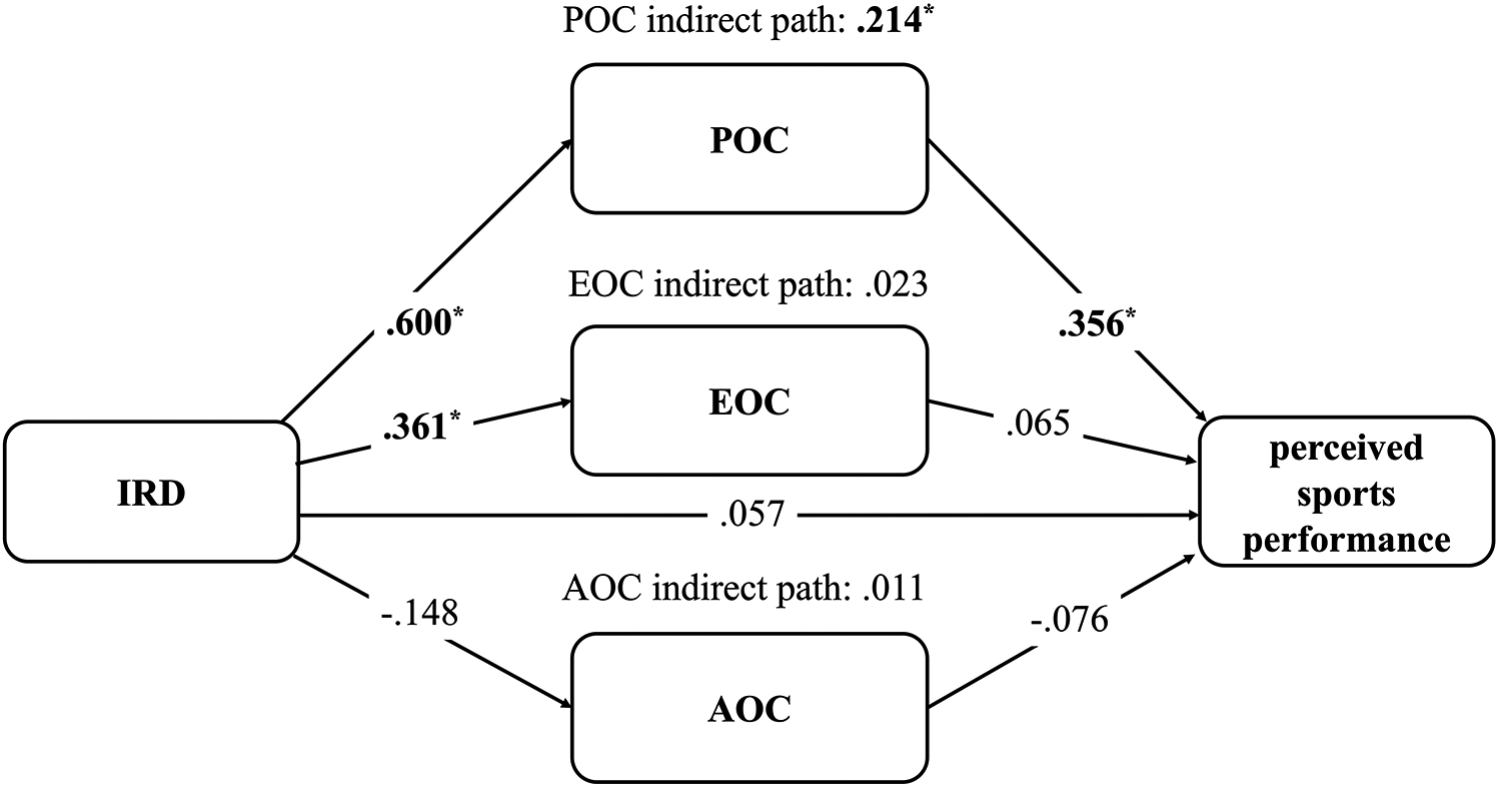
The model of adaptive ruminative disposition and perceived sports performance. Age, sex, and weekly training hours were control variables. IRD, instrumental ruminative disposition; POC, problem-oriented coping; EOC, emotion-oriented coping; AOC, avoidance-oriented coping. All the indexes are standardized regression coefficients (β); ^*^ CI does not include zero.

After controlling for age, sex, and weekly training hours, the indirect association through POC was significantly positive [95% CI (0.420, 0.051)], while EOC [95% CI (0.120, −0.045)] and AOC [95% CI (0.052, −0.017)] were non-significant. Simultaneously, IRD had a non-significant direct association with perceived sports performance [95% CI (0.270, −0.156)]. Therefore, the model indicated that only POC completely mediated the relationship between IRD and perceived sports performance (Figure 2).

Specifically, regarding each of the associations within the model, IRD was significantly positively associated with both POC [95% CI (0.754, 0.447)] and EOC [95% CI (0.540, 0.182)], and not AOC [95% CI (0.041, −0.336)]. Among the coping strategies, only POC was significantly positively associated with perceived sports performance [95% CI (0.635, 0.077)], while EOC [95% CI (0.306, −0.176)] and AOC [95% CI (0.010, −0.252)] were not (Figure 2).

#### 3.2.4 The *post hoc* power analysis

Extracting the standard coefficients from the significant results, the estimated power for the ERD, MRD, and IRD models in the indirect association of POC was 0.78, 0.59, and 0.37, respectively. None of them reached the adequate power of 0.80.

## 4 Discussions

Our cross-sectional study focused on young elite athletes in Hong Kong, aiming to investigate the relationship between multi-dimensional ruminative dispositions and perceived sports performance. We examined whether coping strategies serve as potential connections in this relationship using parallel mediation analysis. The main findings revealed that both ERD and MRD were negatively associated with perceived sports performance indirectly via POC, and IRD demonstrated a positive indirect association via POC.

Our findings align with previous empirical research studies demonstrating a relationship between rumination and specific sports performance and performance-related outcomes. Scott et al. (10) found a positive relationship between ruminative disposition characterized by negative emotion and unforced errors in tennis players. Similarly, Kinrade et al. (9) found that athletes who tended to ruminate excessively on past poor decisions exhibited lower accuracy in high-complexity basketball passing tasks under stress. Furthermore, Josefsson et al. (8) highlighted a negative association between brooding rumination and difficulties in coping with competition stress. Notably, our study employed a more comprehensive perceived approach to measuring sports performance, which not only effectively standardized the performance across various specialties (51) but also integrated both training and competition performance into a single overall measurement to mitigate the risk of invalid responses compared to multi-item approaches (54). We also adopted a multi-dimensional framework within the classification of ruminative dispositions (25, 26). This framework considers various facets of rumination, including the established distinction between “brooding rumination” and “reflective rumination” (19, 20), and further incorporates the distinction between abstract (i.e., focusing on attribution) and concrete (i.e., focusing on problem-solving) reflective rumination (20, 23). The framework allowed for a more nuanced examination of the relationship between ruminative dispositions and sports performance.

The negative indirect association between maladaptive ruminative dispositions (i.e., ERD and MRD) and perceived sports performance through POC could be explained by Baumeister et al.'s (70) strength model of self-control. This model proposes that individuals have limited cognitive resources and engaging in any active control psychological process depletes these resources, ultimately leading to difficulty in engaging in any further active cognitive activities (71). To elaborate, individuals who engage in rumination within the abstract aspect or focus on negative emotions, often characterized by their unwanted and intrusive nature, may consume a significant amount of cognitive resources (72, 73). Furthermore, POC involves active control processes (74), which are effective in dealing with the adversities encountered during competitions or training (33, 34). Consequently, athletes with higher ERD or MRD may be unable to effectively utilize POC to deal with these adversities, potentially leading to decreased perceived sports performance. In addition, according to the Goal Progress Theory (4), rumination seems to be a process that reminds individuals of the problems that hinder their progress toward their goals (5, 6). However, if individuals become excessively focused on these problems or their associated negative feelings during rumination, it may subsequently impair motivation or even induce depression (75). An empirical study indicated that individuals who habitually ruminated on the negative aspects of their experience were more likely to have lower motivation for daily activity participation (76). Similarly, in a sports context, Michel-Kröhler and Berti (77) found that difficulties in maintaining goal pursuit after failure were linked to a disposition to dwell on negative emotions or fixate on problems. Therefore, athletes with higher ERD or MRD may experience reduced motivation to adopt active coping strategies to overcome obstacles, thereby perceiving worse performance. Along with cognitive resources and motivation, a recent meta-analysis indicated that rumination had a strong positive relationship with depression (78). Moreover, Visser et al. (79) found that clinical individuals with more severe depression were less likely to utilize POC. Thus, depression may be a potential factor that mediates the relationship between ruminative dispositions, coping strategies, and performance; however, further exploration of this issue is needed.

A potential explanation of the positive indirect association between IRD and perceived sports performance though POC is presented below. In contrast to maladaptive ruminators whose rumination processes following negative experiences focus on abstract and emotionally laden content (3), the analytical rumination hypothesis suggests that rumination emphasizing problem-solving is beneficial for emotional adaptation and problem resolution (24, 80). Moreover, rumination focused on problem-solving is more likely to be reflected upon and rehearsed to avoid mistakes after setbacks (81). To elaborate, rumination that focuses on problem-solving can be regarded as mental imagery (82–84). According to the functional equivalence model, engaging in mental imagery of problem-solving in their consciousness enables individuals to more clearly process similar problems when they are encountered in the future (85). Additionally, recent studies in the sports context also indicated that athletes engaging in sports-related mental imagery had enhanced sports performance in various specialties (86–88). Therefore, the problem-solving-oriented rumination pattern (e.g., IRD) may be a form of mental imagery that prompts athletes to more swiftly adopt POC to handle stressors in future competitions, thereby avoiding excessive stress that could compromise performance.

In contrast to the role of POC between ruminative disposition and perceived performance, the indirect associations between ruminative dispositions (i.e., ERD, MRD, and IRD) and perceived sports performance through EOC and AOC were non-significant. The non-significant indirect association of EOC could suggest that not all EOC leads to peak performance. EOC targets alleviating emotion associated with the stressor (31, 32) and can be further distinguished into self-regulation and interpersonal regulation (36). Previous studies mostly found a positive effect of self-regulation on performance, such as mindfulness (38, 40), distraction (37), and reappraisal (37, 39). Similarly, Tamminen et al. (36) also indicated that only self-regulation enhanced performance outcomes, whereas interpersonal regulation (e.g., seeking emotional support from others) did not. Additionally, inappropriate self-regulation (i.e., suppression) could even have a negative effect on sports performance (89). This suggests that only some EOC is effective in enhancing sports performance, which may explain the non-significant association in our study. Furthermore, the non-significant indirect association of AOC might be attributable to the elite athlete sample. Poulus et al. (90) suggested that elite athletes regarded AOC as a more ineffective strategy than POC and EOC, therefore AOC may be less likely to be adopted when coping with stressors. Our findings may align with this perspective, with the sample characteristics potentially masking the relationship between AOC and other variables.

Some extra findings that emerged in our findings revealed that there were negative indirect associations between maladaptive ruminative dispositions (i.e., ERD and MRD) and perceived sports performance through POC, while negative direct associations between maladaptive ruminative dispositions and perceived sports performance were also observed. This partial mediation suggests that the association between maladaptive rumination and perceived sports performance may involve other factors. For instance, from the perspective of lifestyle habits, the frequency of pre-sleep rumination negatively predicts subjective and objective sleep quality (91–93). Additionally, sleep quality is positively correlated with sports performance (94). Therefore, for future investigations into the connections between maladaptive rumination and sports performance, it is recommended to adopt a more diverse and comprehensive perspective considering factors such as lifestyle habits. Additionally, we found a high correlation between ERD and MRD in our study, suggesting that these two forms of rumination may be highly interrelated or even partially overlapping constructs. This finding aligns with previous research which demonstrated that athletes with higher levels of brooding were unable to stop thinking about competition-related problems (77). Future studies should continue refining the frameworks of ruminative dispositions within the sports context to further validate the relationship between ruminative dispositions, coping strategies, and sports performance.

## 5 Limitations

Although our study has proposed a theoretically grounded mediation model with statistical significance, its cross-sectional nature limits the inference of causal relationships (95). To strengthen the causal evidence for the model structure, future research should employ prospective or intervention designs. Furthermore, the small sample size (power < 0.80 in the parallel mediation analysis) presents a challenge for ensuring the reliability of SEM (96–98). Therefore, the results should be interpreted with caution. Future research studies with sufficient participants should conduct structural equation modeling (SEM) to combine three models in this study to provide more comprehensive evidence with reliable model fit (99) and confirm measurement invariance (e.g., age) (100) or cross-contextual consistency (e.g., general vs. sports; or training vs. competition) (101) to ensure the validity of the scales employed in this study.

Additionally, there is a difference in ruminative dispositions between Eastern and Western cultures. Previous studies showed Asians tended to ruminate more on emotions (102, 103), but the association with maladaptive outcomes was weaker (102). Interestingly, despite these differences, both cultures demonstrated an equal degree of ruminative dispositions that focus on preventing future failures (104). This raises concerns about applying our findings to Western athletes, particularly regarding the ERD model. Conversely, the IRD model, which may be less influenced by cultural variations in rumination, could potentially hold better generalizability for Western populations.

## 6 Implications and future directions

Our study has illustrated the association between ruminative dispositions, coping strategies, and performance through the lens of multi-dimensional rumination. The findings provided a different view from past studies and indicated that not only are effective coping strategies important for sports performance but also the proper ruminative response when the expectation does not match the performance. Specifically, ERD and MRD may be risk factors associated with decreased perceived sports performance, while IRD may have the opposite relationship with perceived sports performance. This insight suggests that practitioners, such as coaches and sports psychology consultants, should prioritize addressing athletes' ruminative dispositions.

A previous study has suggested that a mindfulness intervention is an effective strategy for reducing athletes' ERD and enhancing sports performance (27); furthermore, a mindfulness intervention that emphasizes self-compassion may be more effective in reducing ERD (12, 14). However, interventions aimed at diminishing MRD and enhancing IRD in the sports context remain unclear. Future research could draw insights from rumination-focused cognitive-behavioral therapy (RF-CBT) from clinical psychology, which aims to shift individuals' focus from abstract and non-constructive content during rumination to concrete and constructive content (3). Moreover, randomized controlled trials have demonstrated its positive effects in reducing abstract ruminative dispositions and clinical symptoms in depression (105). Hence, practitioners and scholars could also cooperate to build upon this foundation to develop psychological skill training with the potential to enhance IRD and reduce ERD and MRD in athletes.

## 7 Conclusion

This study applies the theoretical framework of multi-dimensional rumination to the sports context. Athletes with higher maladaptive ruminative dispositions (i.e., ERD and MRD) are less prone to using problem-orientation coping strategies to deal with stressful events, which may potentially impair their perceived sports performance. Conversely, athletes with higher adaptive ruminative disposition (i.e., IRD) are more proactive in dealing with the problems causing stress, which may enhance perceived sports performance. This suggests that while rumination is a common cognitive process in sports, it does not inherently have a negative relationship with performance. Rather, the outcome of rumination may vary depending on its focus. Specifically, concentrating on “how” to prevent similar problems in the future may be more beneficial for athletic performance than focusing on “why” these problems occurred or dwelling on negative emotions. The proposed model not only validates the theoretical framework of multi-dimensional rumination in sports psychology but also offers a preliminary model outlining the potential impact mechanisms of ruminative dispositions on athletic performance. It serves as a reference for future researchers and practitioners, including athletes, coaches, and sports psychology consultants.

## Data Availability

The raw data supporting the conclusions of this article will be made available by the authors, without undue reservation.
