# Supplementary material for: Relationship between ruminative dispositions and perceived sports performance in young elite athletes in Hong Kong: the role of problem-oriented coping strategies
Source: Front Sports Act Living. 2025 Feb 28;7:1513277. doi: 10.3389/fspor.2025.1513277 (PMC11906345; doi:10.3389/fspor.2025.1513277)
Supplement: Supplementary file 1 [file Supplementaryfile1.docx]

**Supplementary material**

Table 1. Correlation matrix among the three ruminative dispositions

| Ruminative dispositions | ERD | MRD |
| --- | --- | --- |
| MRD | .852^***^ |  |
| IRD | −.099 | −.084 |

Note. ERD is emotion-focused ruminative disposition, and MRD is meaning-searching ruminative disposition, IRD is instrumental ruminative disposition. ^*^ *p* < .05. ^**^ *p* < .01. ^***^ *p* < .001.

Table 2. Sex difference in ruminative dispositions, Coping strategies and perceived sports performance

| Variables |  | | Sex | |  | | *t* | *df* | *p* |
| --- | --- | --- | --- | --- | --- | --- | --- | --- | --- |
|  | Male (*n* = 55) | | | Female (*n* = 56) | | |  |  |  |
|  | *M* (*SD*) | 95% CI | | *M* (*SD*) | | 95% CI |  |  |  |
| Ruminative dispositions |  |  | |  | |  |  |  |  |
| ERD | 2.270 (0.816) | [2.486, 2.054] | | 2.440 (0.847) | | [2.669, 2.211] | –1.074 | 109 | .285 |
| MRD | 2.143 (0.814) | [2.358, 1.928] | | 2.400 (0.781) | | [2.605, 2.195] | –1.668 | 109 | .098 |
| IRD | 3.673 (0.659) | [3.847, 3.499] | | 3.568 (0.547) | | [3.711, 3.425] | 0.915 | 109 | .362 |
| Coping strategies |  |  | |  | |  |  |  |  |
| POC | 4.601 (0.875) | [4.832, 4.370] | | 4.643 (0.783) | | [4.848, 4.438] | –0.264 | 109 | .792 |
| EOC | 4.661 (1.020) | [4.931, 4.391] | | 4.819 (0.963) | | [5.071, 4.567] | –0.844 | 109 | .401 |
| AOC | 2.333 (0.985) | [2.593, 2.073] | | 2.354 (0.941) | | [2.600, 2.108] | –0.114 | 109 | .909 |
| Perceived sports performance | 6.160 (1.813) | [6.639, 5.681] | | 6.140 (1.600) | | [6.559, 5.721] | 0.064 | 109 | .949 |

Note. ERD is emotion-focused ruminative disposition, and MRD is meaning-searching ruminative disposition, IRD is instrumental ruminative disposition; POC is problem-oriented coping, EOC is emotion-oriented coping, and AOC is avoidance-oriented coping; ^*^ *p* < .05. ^**^ *p* < .01. ^***^ *p* < .001; Value expressed as means (standard deviation) for the variable.

Table 3. Developmental stages difference in ruminative dispositions, Coping strategies and perceived sports performance

| Variables | Developmental stage | | | | *t* | *df* | *p* |
| --- | --- | --- | --- | --- | --- | --- | --- |
|  | Adolescent (n = 56) | | Adolescent (n = 55) | |  |  |  |
|  | *M* (*SD*) | 95% CI | *M* (*SD*) | 95% CI |  |  |  |
| Ruminative dispositions |  |  |  |  |  |  |  |
| ERD | 2.495 (0.881) | [2.726, 2.264] | 2.214 (0.763) | [2.416, 2.012] | 1.792 | 109 | .076 |
| MRD | 2.347 (0.846) | [2.569, 2.125] | 2.192 (0.759) | [2.393, 1.991] | 1.014 | 109 | .313 |
| IRD | 3.657 (0.626) | [3.821, 3.493] | 3.582 (0.582) | [3.736, 3.428] | 0.656 | 109 | .513 |
| Coping strategies |  |  |  |  |  |  |  |
| POC | 4.658 (0.924) | [4.900, 4.416] | 4.585 (0.720) | [4.775, 4.395] | 0.463 | 109 | .644 |
| EOC | 4.696 (1.115) | [4.988, 4.404] | 4.786 (0.853) | [5.011, 4.561] | –0.475 | 102.893 | .636 |
| AOC | 2.470 (0.994) | [2.730, 2.210] | 2.251 (0.912) | [2.492, 2.010] | 1.408 | 109 | .162 |
| Perceived sports performance | 6.390 (1.569) | [6.801, 5.979] | 5.910 (1.808) | [6.388, 5.432] | 1.506 | 109 | .135 |

Note. IRD is instrumental ruminative disposition, ERD is emotion-focused ruminative disposition, and MRD is meaning-searching ruminative disposition; POC is problem-oriented coping, EOC is emotion-oriented coping, and AOC is avoidance-oriented coping; ^*^ *p* < .05. ^**^ *p* < .01. ^***^ *p* < .001; Value expressed as means (standard deviation) for the variable; The cutoff-point between adolescent and adult is above 19.

Figure 1. The correction formula of multiple correlation

Note. “adj” represents the value has been adjusted. “k” is the numbers of correlation coefficients.

In our primary correlation analysis, each mediator was correlated with three independent variables (i.e., ERD, MRD, and IRD) and one dependent variable (i.e., perceived sports performance), resulting in k = 4.

In our exploratory correlation analysis, which examined the relationships among ERD, MRD, and IRD, resulting in k = 3.
